# The NKG2D Ligands RAE-1δ and RAE-1ε Differ with Respect to Their Receptor Affinity, Expression Profiles and Transcriptional Regulation

**DOI:** 10.1371/journal.pone.0013466

**Published:** 2010-10-19

**Authors:** Oriane Cédile, Natalia Popa, Frédéric Pollet-Villard, Nicolas Garmy, El Chérif Ibrahim, José Boucraut

**Affiliations:** 1 CRN2M, CNRS UMR 6231, Université de la Méditerranée, Université Paul Cézanne, Faculté de Médecine, Marseille, France; 2 NICN, CNRS, UMR 6184, Université de la Méditerranée, Faculté de Médecine, Marseille, France; Karolinska Institutet, Sweden

## Abstract

**Background:**

RAE-1 is a ligand of the activating receptor NKG2D expressed by NK cells, NKT, γδT and some CD8^+^T lymphocytes. RAE-1 is overexpressed in tumor cell lines and its expression is induced after viral infection and genotoxic stress. We have recently demonstrated that RAE-1 is expressed in the adult subventricular zone (SVZ) from C57BL/6 mice. RAE-1 is also expressed *in vitro* by neural stem/progenitor cells (NSPCs) and plays a non-immune role in cell proliferation. The C57BL/6 mouse genome contains two *rae-1* genes, *rae-1δ* and *rae-1ε* encoding two different proteins. The goals of this study are first to characterize the *in vivo* and *in vitro* expression of each gene and secondly to elucidate the mechanisms underlying their respective expression, which are far from known.

**Principal Findings:**

We observed that Rae-1δ and Rae-1ε transcripts are differentially expressed according to tissues, pathological conditions and cell lines. Embryonic tissue and the adult SVZ mainly expressed Rae-1δ transcripts. The NSPCs derived from the SVZ also mainly expressed RAE-1δ. The interest of this result is especially related to the observation that RAE-1δ is a weak NKG2D ligand compared to RAE-1ε. On the contrary, cell lines expressed either similar levels of RAE-1δ and RAE-1ε proteins or only RAE-1ε. Since the protein expression correlated with the level of transcripts for each *rae-1* gene, we postulated that transcriptional regulation is one of the main processes explaining the difference between RAE-1δ and RAE-1ε expression. We indeed identified two different promoter regions for each gene: one mainly involved in the control of *rae-1δ* gene expression and the other in the control of *rae-1ε* expression.

**Conclusions/Significance:**

RAE-1δ and RAE-1ε differ with respect to their function and the control of their expression. Immune function would be mainly exerted by RAE-1ε and non-immune function by RAE-1δ.

## Introduction

RAE-1 is a ligand of the activating receptor NKG2D expressed by NK cells, NKT, γδT and some CD8^+^T lymphocytes [1]. RAE-1 protein is a MHC (major histocompatibility complex) class I-related molecule composed of 2 domains, α1 and α2 forming a groove similar to the MHC class I molecules but which is too small to present a peptide [2]. Five RAE-1 proteins have been described which are encoded by 5 different *rae-1* genes: *α, β, γ, δ* and *ε.* The genome of the BALB/C mouse strain has four *rae-1* genes: *α*, *β*, *γ* and *δ*, the 129/sv, 3 genes: *α*, *β* and *γ* and the C57BL/6, only 2 genes: *δ* and *ε*.

Rae-1 transcripts have been first isolated from mouse embryonic carcinoma F9 cell line among retinoic acid inducible clones [3]. RAE-1 is overexpressed in tumor cell lines [1], [4], [5] and its expression is induced after viral infection [6], [7] and genotoxic stress [8]. RAE-1 is constitutively expressed during embryogenesis, mainly in the brain [9] whereas, in adult BALB/c mice, Rae-1 transcripts are not detected except in the adult spleen and liver where very low amounts are found [1]. Higher levels of Rae-1 transcripts are detected in the liver of C57BL/6 mice and interestingly this expression is circadianly regulated [10]. We have recently demonstrated that, in C57BL/6 mice, RAE-1 expression persists in one of the main area of adult neurogenesis, the subventricular zone (SVZ) [11]. Moreover, neural stem/progenitor cells (NSPCs) derived from postnatal mouse SVZ express RAE-1 and the expression of RAE-1 is lost after neural differentiation. So far, RAE-1 is known for its immune functions. We have demonstrated that RAE-1 also exerts a non-immune function in cell proliferation of NSPCs [11].

The molecular mechanisms controlling the expression of each *rae-1* gene are far from known.

Nausch N. et al. showed that embryonic cell lines from C57BL/6 mice lacking JUNB, a subunit of the AP-1 complex, expressed higher level of RAE-1ε than control cells lines whereas the expression of RAE-1δ was not enhanced [12]. This study suggests for the first time that the molecular mechanisms controlling the expression of *rae-1δ* and *rae-1ε* are different. The goals of our study were thus to characterize the *in vivo* and *in vitro* expression of each gene and to elucidate the mechanisms underlying their respective expression.

We observed that Rae-1δ and Rae-1ε transcripts are differentially expressed according to tissues, pathological conditions and cell lines. SVZ and neurosphere cells expressed mainly RAE-1δ. Interestingly, RAE-1δ is a poor NKG2D ligand compared to RAE-1ε. Because RAE-1δ and RAE-1ε protein expression level *in vitro* correlated with the level of their respective transcripts, we postulate that transcriptional regulation is one of the main mechanisms explaining the difference between Rae-1δ and Rae-1ε transcripts expression. We indeed identified two different promoter regions for each gene, one upstream of exon 1, mainly involved in the control of *rae-1ε* gene expression and a second one, upstream of a previously unknown exon, named exon 3b, mainly involved in the control of *rae-1δ* gene expression.

## Results

### RAE-1δ and RAE-1ε differ with respect to their expression profile and NKG2D affinity

**Figure 1:**
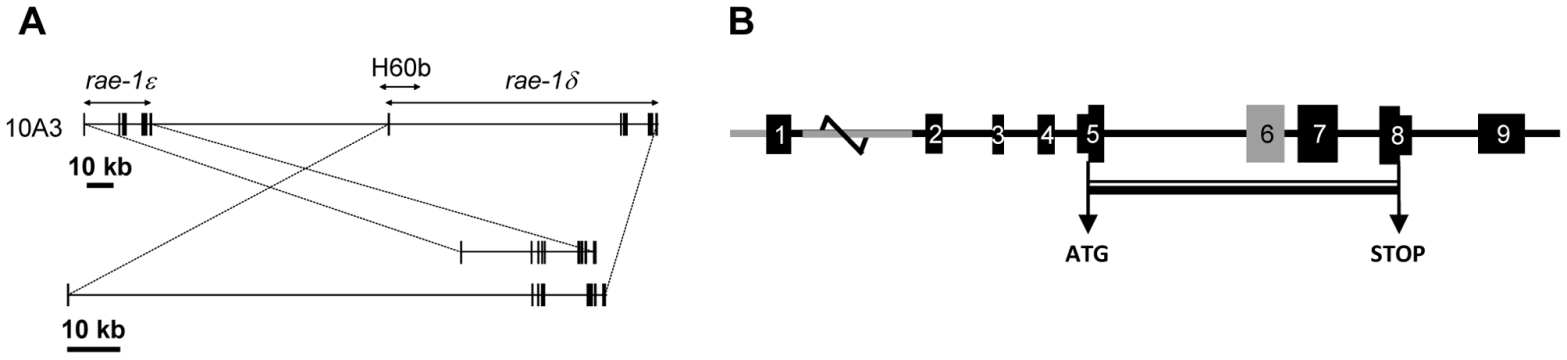
Schematic representation of *rae-1* genes. A, Exon/intron structure and localization of *rae-1δ* and *rae-1ε* genes on the 10 A3 chromosomal region. B, Representation of *rae-1* genes. The most important differences between *rae-1δ* and *rae-1ε* are colored in grey.

*In silico* study of *rae-1δ* and *rae-1ε* genes revealed that they are located on chromosome 10 band A3 in the C57BL/6 genome, adjacent but separated by 89.5 kb (Fig. 1A). This region also contains genes encoding other NKG2D ligands like MULT1, H60c (10 A1) and H60b (10 A3) [13]. Rae-1δ and Rae-1ε GenBank cDNA sequences contain 6 full exons (NM_020030 and NM_198193 respectively). However, analysis of the available genomic sequences, based on the comparison with the Rae-1α cDNA sequence (NM_009016) described by Nomura and coll. [9], allowed us to identify 3 additional exons in both *rae-1δ* and *rae-1ε* genes. The figure 1B represents the *rae-1* genes. The coding sequence starts in the middle of exon 5 and ends in the middle of exon 8. Exon 5 encodes the leader sequence, exons 6 and 7 encode the extracellular α1 and α2 domains respectively and exon 8 the GPI anchor. The main differences between the two genes lie within the sequences of exon 6 and upstream of exon 1 but also in the length of intron 1. These differences allowed us to design PCR primers to distinguish the expression of Rae-1δ and Rae-1ε transcripts.

**Figure 2:**
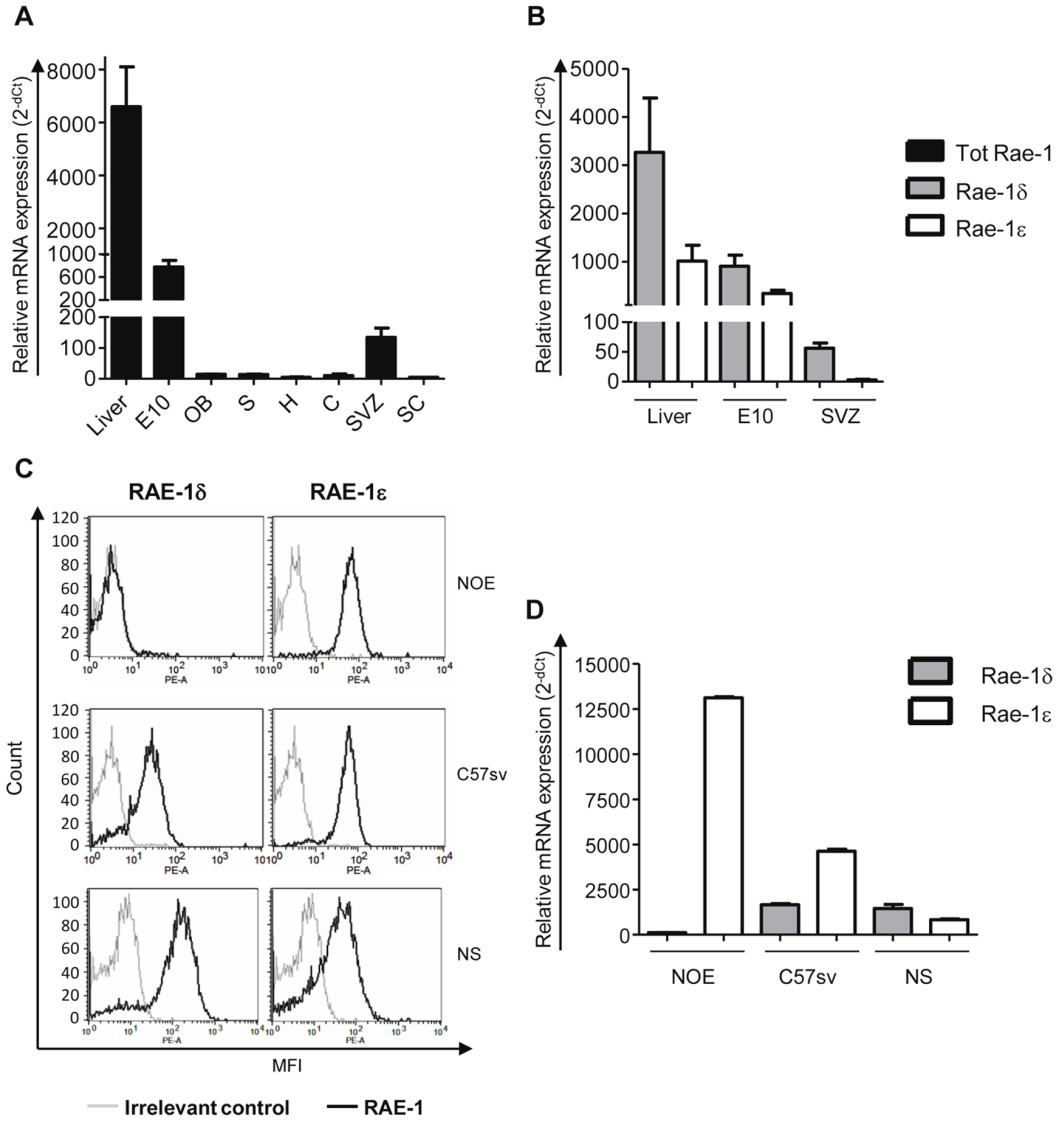
Expression of Rae-1 transcripts in C57BL/6 tissues and cell lines. A and B, Quantification of all Rae-1, Rae-1δ and Rae-1ε transcripts in healthy tissues: 10-days-old embryo (E10), liver and different parts of the adult central nervous system: olfactory bulbs (OB), subventricular zone (SVZ), spinal cord (SC), striatum (S), cerebellum (C) and hippocampus (H). Results were expressed as mean of 3 analyses +/− SEM relatively to GAPDH as endogenous control. C, Expression of RAE-1δ and RAE-1ε proteins analyzed on NOE and C57sv cell lines and neurosphere cells by flow cytometry after staining with anti-RAE-1δ or -RAE-1ε antibodies and PE-labeled anti-rat immunoglobulins. D, Expression of Rae-1δ and Rae-1ε transcripts by NOE and C57sv cell lines and neurosphere cells, quantified by qPCR.

We analyzed the respective expression level of Rae-1δ and Rae-1ε transcripts *in vivo*. We confirmed the presence of Rae-1 transcripts in embryonic tissues and detected Rae-1 transcripts in adult tissues (Fig. 2A). Rae-1 was highly expressed in the liver and undetectable in numerous parts of the brain except in the SVZ, the main area of neurogenesis, where Rae-1 was weakly but significantly detected. Both Rae-1δ and Rae-1ε transcripts were expressed in liver and embryonic tissues (Fig. 2B) with a preferential expression of Rae-1δ. In the SVZ, mostly Rae-1δ transcripts were expressed (Fig. 2B). We then compared the respective expression of RAE-1δ and RAE-1ε transcripts and proteins in C57BL/6 cell lines and neurospheres obtained from the SVZ (Fig. 2C). Preferential expression of Rae-1ε transcripts was observed in the C57sv cell line. The NOE olfactory epithelial cell line only expressed RAE-1ε and neurosphere cells preferentially expressed RAE-1δ as the SVZ. Using specific RAE-1ε and RAE-1δ antibodies we observed that the pattern of protein expression is similar to transcripts (Fig. 2D). Thus, transcriptional regulation is probably the main process underlying the differential expression of RAE-1δ and RAE-1ε.

**Figure 3:**
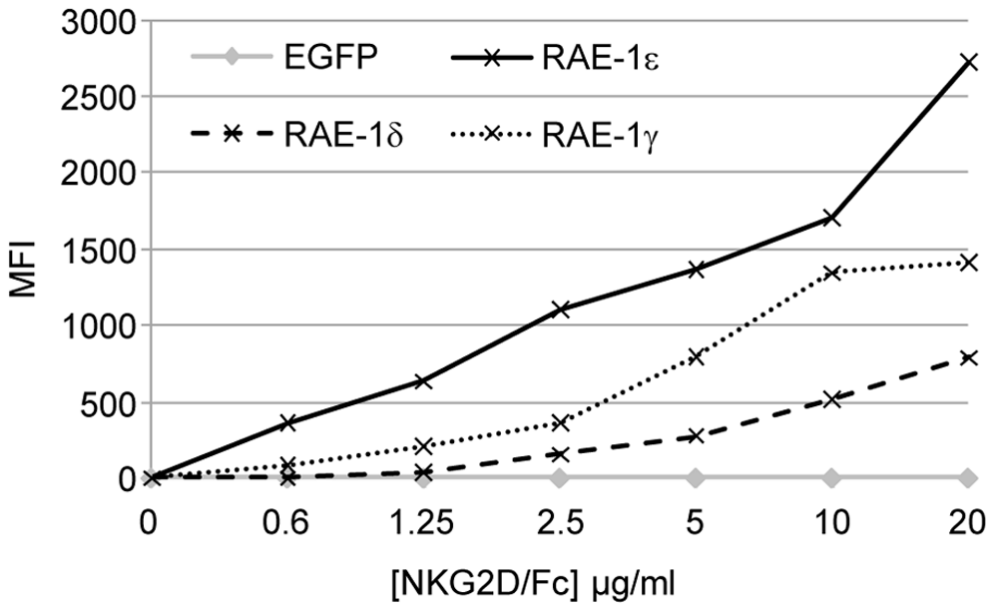
Comparison of NKG2D affinity for RAE-1 proteins. HEK cell line was cotransfected with plasmids coding for EGFP as a transfection efficiency control and either RAE-1δ, RAE-1ε or RAE-1γ. After 3 days of culture, cells were incubated with increasing concentrations of NKG2D/Fc and PE-labeled anti-human IgG-Fc. Results were expressed as: (PE MFI measured on EGFP positive cells) - (PE MFI measured on EGFP negative cells).

We then compared by flow cytometry the binding of NKG2D/Fc on HEK cells transfected with plasmids encoding RAE-1δ, RAE-1ε or RAE-1γ (Fig. 3). NKG2D bound to RAE-1ε and RAE-1γ at low concentration (0.6 µg/ml) whereas binding to RAE-1δ was only detected at 2.5 µg/ml. At each concentration, NKG2D/Fc binding to RAE-1ε was stronger than to RAE-1γ and NKG2D/Fc binding to RAE-1γ was stronger than to RAE-1δ (Fig. 3). RAE-1δ is thus a poor NKG2D ligand.

In conclusion, we showed that RAE-1δ and RAE-1ε are two different proteins and that their expression is differentially regulated. We thus investigated the mechanisms controlling the expression of each gene.

### Rae-1δ and Rae-1ε transcription is under the control of two distinct promoter regions

We first performed an *in silico* study of *rae-1δ* and *rae-1*ε sequences to predict their promoters. We identified two promoter regions for each gene, one localized upstream of exon 1 and a second, upstream of exon 4 (Gene2promoter program, Genomatix software).

**Figure 4:**
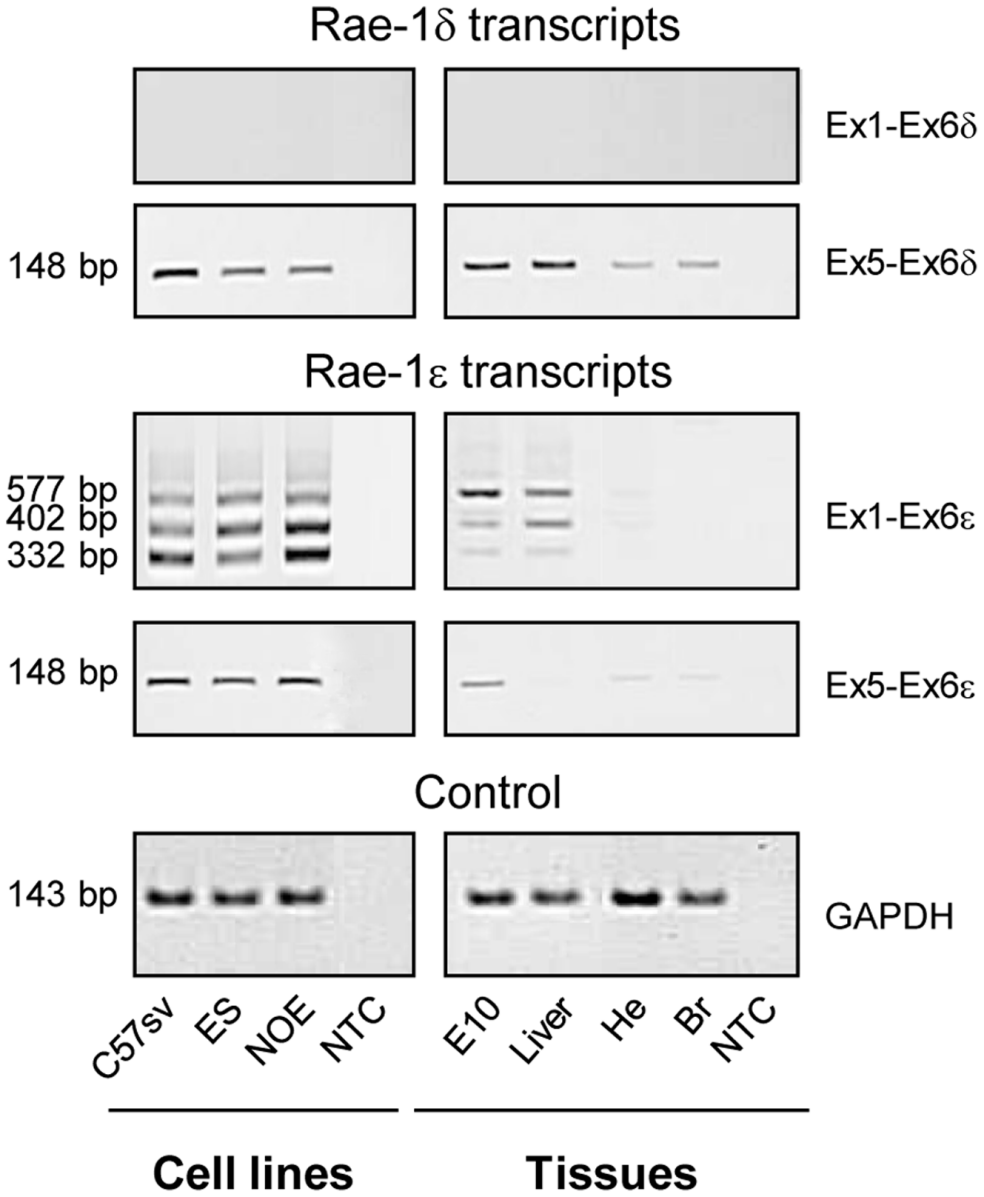
Characterization of Rae-1δ and Rae-1ε transcripts. A, RT-PCR analysis of Rae-1δ transcripts using an exon 1-exon 6δ primer pair or an exon 5-exon 6δ primer pair and of Rae-1ε transcripts using an exon 1-exon 6ε primer pair or an exon 5-exon 6ε primer pair (see schematic in Fig. 6). We compared the PCR products obtained from cell lines (C57sv, ES, NOE) and healthy tissues (10-days-old embryo (E10), liver, heart (He) and brain (Br)); NTC, non template control.

We then analyzed all Rae-1 transcripts possibly generated by these promoters in C57BL/6 cell lines and tissues using RT-PCR and agarose gel analysis. The Ex5-Ex9 primer pair does not discriminate the Rae-1δ from the Rae-1ε transcripts (data not shown). Using these primers, we detected a single product. We sequenced the liver PCR product obtained with those primers and observed the superimposition of two sequences, one corresponding to Rae-1δ and the other one less represented corresponding to Rae-1ε (Figure S1). The figure 4 illustrates the results obtained with Ex1-Ex6δ and Ex1-Ex6ε pairs that are gene-specific and may reflect the activity of the putative promoter upstream of exon 1. Using an exon 5 and exon 6δ primer pair, Rae-1δ transcripts were detected in all tested conditions. However, we failed to detect Rae-1δ transcripts using the primer targeting exon 1 (Fig. 4, upper panels). We only detected exon 1-containing Rae-1δ transcripts in the NOE cell line (referenced FJ594066 in GenBank) after treatment with actinomycin D and cycloheximide which stabilize RNA (data not shown). Finally, the primer pair targeting exon 1 and exon 6 of Rae-1ε, allowed the detection of three main PCR products, one full length and two alternative splicing products of either exon 2 alone (402 bp) or exon 2 and exon 3 (336 bp) (Fig. 4, middle panels) which were sequenced and referenced in GenBank, FJ 594065, FJ594067 and FJ594068 respectively. These observations suggested that transcription of *rae-1* genes is under the control of two different promoter regions, one upstream of exon 1 controlling the expression of Rae-1ε, and the second, upstream of exon 5 mainly involved in the expression of Rae-1δ.

**Figure 5:**
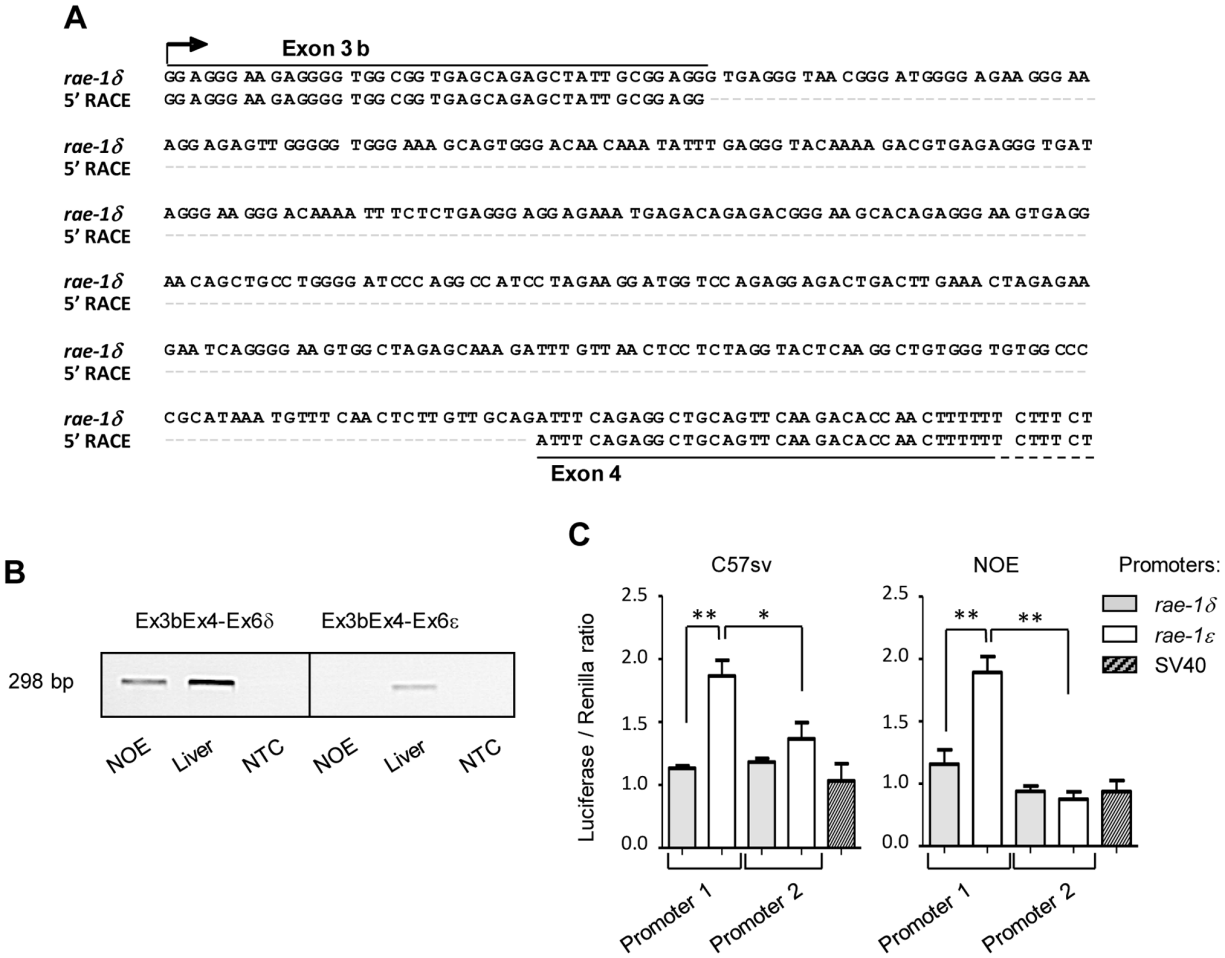
Localization of the *rae-1δ* transcriptional start site and activity of *rae-1δ* and *rae-1ε* promoters. The 5′ RACE experiment was performed on enriched polyA+ RNA isolated from liver. A, The 5′ RACE product was sequenced and aligned with the *rae-1δ* genomic sequence. B, Detection in the liver and NOE cell line of Rae-1δ and Rae-1ε transcripts under the control of promoter 2 using a sense primer overlapping the exon 3b and the exon 4. C, NOE and C57sv cell lines were co-transfected with the luciferase reporter vector pGL3 Basic containing the different Rae-1 promoters or the pGL3-C vector containing the SV40 promoter and the pRL-null vector expressing *renilla* luciferase in order to assess the efficiency of transfection and to normalize the activity of the firefly luciferase. Luciferase activity was measured on each triplicate wells 48 h after transfection. The results are expressed as mean of 3 experiments in triplicate +/− SEM of firefly/*renilla* luciferase activity ratio for each condition. The assays of luciferase activity were analyzed with a MANOVA test (*: p<0.01 and **: p<0.001).

To validate this hypothesis, we performed 5′RACE experiments starting from either exon 3 or exon 6δ. The sequencing of the product starting from exon 6δ revealed a transcription start site 41 nucleotides upstream of exon 4 and the presence of an additional exon that we named exon 3b (Fig. 5A), also found in the *rae-1ε* gene. Accordingly, former exon 3 was renamed exon 3a (Fig. 5A). Sequencing of the products starting from exon 3a confirmed the presence of a transcription start site 15 nucleotides upstream of exon 1 of *rae-1ε* gene (data not shown) as previously described for *rae-1α* gene [9]. The exon 3b is absent from transcripts initiated at the exon 1. This observation allowed us to test the expression of Rae-1δ or Rae-1ε transcripts under the control of promoter 2 alone, which we called short transcripts. We performed PCR with a forward primer overlapping exon 3b and exon 4, specific for short transcripts, and a reverse primer in exon 6, specific for either Rae-1δ or Rae-1ε (Fig. 5B). We detected Rae-1δ and Rae-1ε short transcripts in the liver. We did not detect any Rae-1ε short transcript in the NOE cell line suggesting that Rae-1ε transcripts are only long transcripts under the control of the promoter 1 in this cell line (Fig. 5B).

We then compared the activity of each promoter regions to the SV40 promoter (pGL3-C). For each gene, we cloned 1,600 bp upstream of exon 1 and 5,646 bp upstream of exon 3b in the pGL3-B vector. After transfection in NOE and C57sv Rae-1-expressing cell lines, each promoter exhibited a high activity. The activity of *rae-1ε* promoter 1 was higher than the activity of *rae-1δ* promoter 1 (Fig. 5C).

In conclusion, we described two promoter regions responsible for the production of several Rae-1δ and Rae-1ε transcripts. We thus questioned the involvement of each promoter in cell lines and tissues, including inflammatory pathological tissues, since Rae-1 expression is enhanced in these conditions [14]. Finally, since Rae-1 expression is circadianly regulated in the liver of C57BL/6 mice, we also addressed the role of each promoter region in this circadian rhythm.

### Quantification of Rae-1δ and Rae-1ε transcript levels under the control of each promoter region in physiological and pathological condition

**Figure 6:**
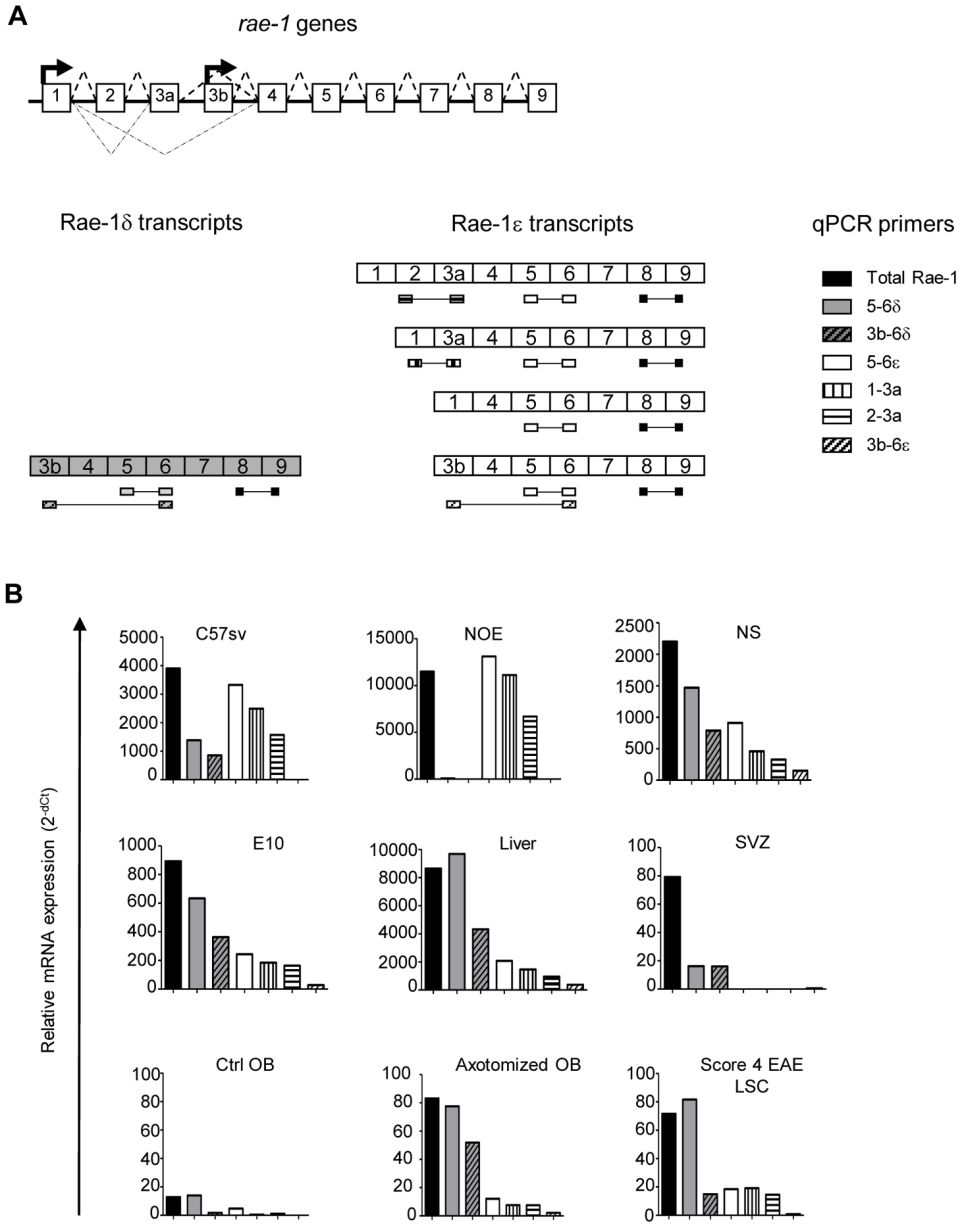
Quantification of all Rae-1 transcripts *in vivo* and *in vitro*. A, Schematic representation of *rae-1* genes with exons (boxes), transcription start sites (bold arrows), intron splicing (bold doted lines) and alternative splicing (thin doted lines). Prediction of all Rae-1δ and Rae-1ε transcripts under the control of either promoter 1 or promoter 2 and position of sets of primers allowing the detection and quantification of each transcript. B, RT-qPCR of each transcript in 10-days-old embryo (E10), subventricular zone (SVZ), liver, olfactory bulbs (OB) from control or axotomized mice, lumbar spinal cord (LSC) from mice suffering EAE and neurospheres (NS), C57sv and NOE cell lines. Results are expressed relatively to GAPDH as endogenous control.

The above precise description of *rae-1δ* and *rae-1ε* genes allowed us to predict several transcripts possibly generated by each promoter which are illustrated in the figure 6A. We designed new sets of primers in order to quantify each Rae-1 predicted transcript in cell lines and tissues. The PCR products obtained with each primer pair are illustrated in figure 6A. Since we detected the recruitment of NKG2D expressing cells in the spinal cord during EAE and in the olfactory bulbs after axotomy (Figure S2), we also investigated the expression of each transcript if these inflammatory conditions.

As shown in figure 6B, the fibroblastic cell line C57sv expressed significant levels of Rae-1δ transcripts whereas the NOE cell line was characterized by a very high expression level of only Rae-1ε. On the contrary, the levels of Rae-1δ were higher than those of Rae-1ε in neurosphere cells, E10 embryonic tissue, liver and pathological tissues. Analysis of the cell lines and tissues confirmed that promoter 2 is mainly involved in the expression of Rae-1δ and that promoter 1 is involved in the expression of Rae-1ε. Even though they were detectable, only few Rae-1ε transcripts included exon 3b. The Ex5-Ex6ε primer pair evaluated all Rae-1ε transcripts whereas the Ex1-Ex3a and Ex2-Ex3a primer pairs indirectly revealed the levels of alternatively spliced products. No clear difference of expression was detected either *in vitro* or *in vivo* between these transcripts. Finally, in the liver and EAE spinal cord, a discrepancy between the results obtained with Ex5-Ex6δ and Ex3b-Ex6δ sets of primers was observed suggesting the presence of at least another type of transcript not detected with these sets of primers. Therefore, we performed additional PCR with primers localized in exon 3a and in exon 6δ. The sequencing of the PCR product showed the absence of the exon3b. This result suggested the presence in the *rae-1δ* gene of another transcription start site upstream of exon 3a.

**Figure 7:**
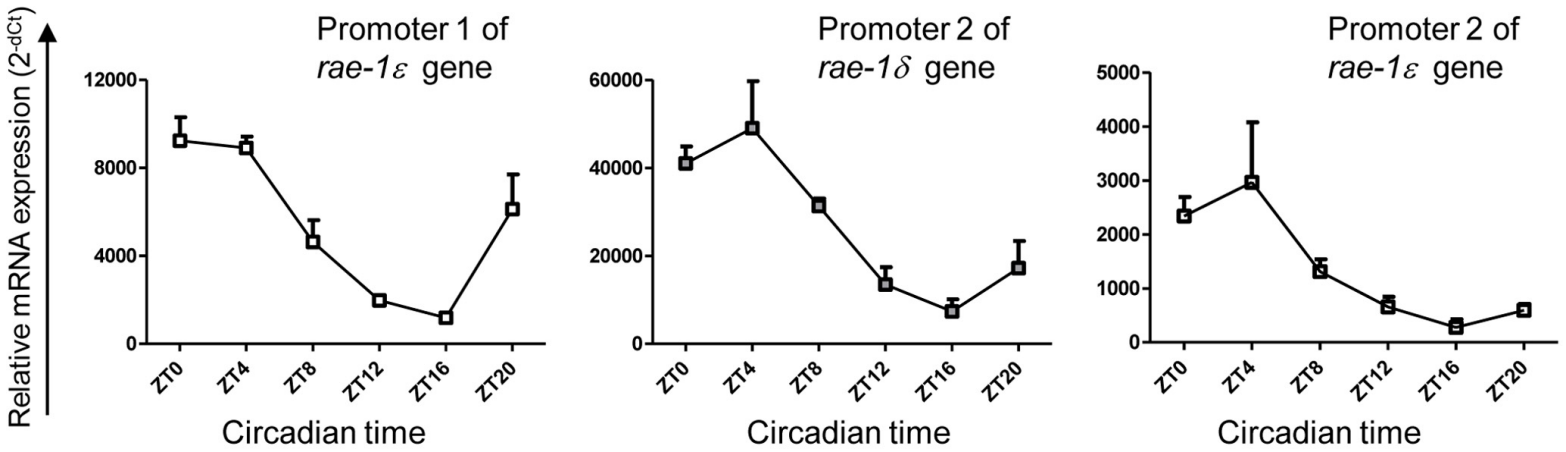
All transcripts are characterized by a circadian rhythm. RT-qPCR were performed on C57BL/6 livers dissected at different Zeitgeber time (ZT) with three sets of primers: exon 1-exon 3a primers which detected the Rae-1ε transcripts under the control of the promoter 1, exon 3b with either exon 6δ or exon 6ε primers allowing the quantification of transcripts under the control of the promoter 2. Results are expressed as mean of 3 livers +/− SEM at each ZT relatively to GAPDH as endogenous control.

Finally, we collected cDNA from C57BL/6 liver at different time points and quantified the transcripts under the control of *rae-1ε* promoter 1, *rae-1ε* promoter 2 and *rae-1δ* promoter 2. We confirmed that Rae-1 transcripts expression is circadianly regulated in the C57BL/6 mice liver [10]. The study showed diurnal rhythm with peak and trough at ZT (Zeitgeber Time) 4 and ZT 16 respectively, for all transcripts (Fig. 7). Because of the differences between the promoters, we can postulate that another transcriptional mechanism is involved in the circadian expression of RAE-1.

## Discussion

We described for the first time the high complexity of the transcriptional expression of Rae-1δ and Rae-1ε in physiological and pathological conditions. Each transcript variants of Rae-1δ and Rae-1ε are differentially expressed according to tissues, pathological conditions and cell lines. This differential expression is mainly explained by the presence of two different promoter regions. The first one, upstream of exon 1, is involved in the expression of *rae-1ε*. The second, mainly involved in the control of *rae-1δ* gene expression, is localized upstream of exon 4 and induces transcripts using a start site upstream of exon 3b. We also brought evidence of another potential start site upstream of exon 3a. The exon 3b is not retained in transcripts using this latter start site.

*In vivo*, even though we could detect the expression of Rae-1ε, this expression was lower than that of Rae-1δ. Conversely, *in vitro*, except for neurosphere culture, cell lines mainly expressed RAE-1ε. Nausch et *al*. also observed that embryonic fibroblastic cell lines established from C57BL/6 mice lacking JUNB, a subunit of the AP-1 complex, express high levels of Rae-1ε whereas the expression of Rae-1δ remained low [12]. *rae-1ε* promoter regions contain more AP-1 sites than those of *rae-1δ* [12]. We hypothesize that culture conditions of immortalized cell lines such as exposition to fetal calf serum, plastic adhesion, high oxygen pressure or stress induction could explain this preferential expression of RAE-1ε [15], [16].

As most genes, *rae-1* genes contain multiple exons/introns and several non-coding exons. The process of pre-mRNA splicing is an essential step in the expression of most genes [17], [18]. For Rae-1ε, we characterized alternative splicing of non-coding exons 2 and 3a but not of coding exons. This process thus did not contribute to the diversification of the RAE-1 protein or the non-sense mediated mRNA decay control mechanism. The biological significance of this alternative splicing remains to be determined.

We confirmed that the expression of Rae-1 transcripts is characterized by a circadian rhythm. The circadian timing is based on a complex network of feedback loops involving transcriptional, post-transcriptional and post-translational events. The main involved transcription factors are PER1, PER2, PER3, CRY1, CRY2, CLOCK/BMAL1 heterodimers and STRA13, a basic helix-loop-helix (bHLH) protein playing a role in the mammalian circadian rhythm regulation by suppressing the CLOCK/BMAL1 activation, but also via protein-protein interactions with other transcription factors including BMAL1 [19]. STRA13 KO mice express lower levels of Rae-1 transcripts than the control mice and their expression is not circadian [10]. Using promoter inspector software, we failed detecting binding sites for STRA13 or CLOCK/BMAL1 in the two *rae-1* promoters. Moreover, we showed that all Rae-1 transcripts, whatever the used promoters, are circadianly expressed. We suggest that regulation of RAE-1 circadian rhythm involves other mechanisms under investigation such as mRNA stabilization or micro-RNAs (miRNAs). Interestingly, the 3′UTR regions of Rae-1δ and Rae-1ε are almost identical and miRNAs have recently been studied in the circadian rhythm of some genes [20]. In addition, it has recently been shown that cellular miRNAs target the 3′UTR of the human NKG2D ligands, MICA and MICB [21], [22].

The interest of our results is especially related to the observation that RAE-1δ is a weak NKG2D ligand compared to RAE-1ε. *In vitro*, neurosphere cells also preferentially express RAE-1δ. This may explain the resistance of neurosphere cells to NK cell cytotoxicity [23] despite the absence of MHC-Ia expression. The mouse cytomegalovirus (MCMV) developed different mechanisms for preventing the anti-viral immune response. In particular, the m152/gp40 glycoprotein of MCMV lowers the membrane expression of RAE-1. Interestingly, the expression of RAE-1ε (as well as α, β and γ) is affected but not the expression of RAE-1δ [24]. As RAE-1δ is a poor NKG2D ligand, we can postulate a more intensive viral effort to regulate RAE-1ε than RAE-1δ. The down-regulation is due to a direct interaction between the m152 and RAE-1 proteins. The retention of RAE-1α, β and γ depends on the amino-acid sequence PLWY (position 19–22), deleted in RAE-1δ and substituted with LPWC in RAE-1ε (Figure S3 A and B) [25]. This sequence lies in an exposed loop connecting the β1 and the β2 strands of α1 domain. The deletion of this sequence might also explain the low affinity of NKG2D/Fc for RAE-1δ, because the tryptophan at position 21 is involved in NKG2D contact [26].

We postulate that immune function is mainly exerted by RAE-1ε and non-immune function by RAE-1δ. RAE-1δ, but not NKG2D are detected in the SVZ. We demonstrated that RAE-1δ exerts a non-immune function on cell proliferation using the neurosphere culture model and a loss of function approach [11]. The mechanism of action remains to be determined. We hypothesize that, as other members of the MHC class-I family, RAE-1 could be a partner for membrane receptors. For example, HFE is a membrane partner of the transferrin receptor [27]. More recently, Mill [28], the closest relative to NKG2D human ligand, MICA, has been described to play roles in nutrient metabolism.

We observed that Rae-1δ and Rae-1ε transcript expression is induced in the spinal cord of EAE mice and olfactory bulbs after axotomy. We also described the recruitment of NK cells expressing NKG2D which exert a neuroprotective role in EAE [29], [30]. It is thus necessary to identify which cells express RAE-1 in the pathological tissues. The first cells possibly expressing RAE-1 are macrophages recruited in the spinal cord after inflammation since LPS treated macrophages express RAE-1 [31]. Other candidates would be neural stem cells since neurogenesis is induced in the spinal cord after EAE [32] and in the olfactory bulbs after axotomy [33].

In conclusion, several studies have improved the understanding of NKG2D receptor-mediated activation, signaling and function [34], [35]. NKG2D recognizes a variety of inducible self-proteins that belong to the non-classical MHC class I family. They include H60 (a, b and c), RAE-1 (α-ε) and MULT1 in mice. Organization and sequence homology of NKG2D ligand family indicate that they are generated by multiple rounds of gene duplication and divergence from an ancestral prototypic gene [35]. These ligands differ in their genomic organization, amino acid sequence, affinity to NKG2D and more importantly in their expression patterns [36]. A rational explanation for the existence of multiple ligands for a single activating receptor NKG2D is still lacking. It depends on a detailed knowledge of their immunobiology and molecular mechanisms regulating the expression of each ligand. We bring herein new information for Rae-1δ and Rae-1ε, showing mainly different functions and transcriptional regulation which may have diverged together.

## Materials and Methods

### Mice

C57BL/6 mice were purchased from Janvier (France). All mice were maintained under pathogen-free conditions in our animal facility (Agreement number C13.055.8). All experiments were performed according to the guidelines of the local ethical committee on animal research.

### Cell culture

Cell lines: We established the NOE cell line from the olfactory epithelium of C57BL/6 mice [37]. The C57sv fibroblast and the HEK 293 epithelial cell line were obtained from the American Type Culture Collection (ATCC). The cell lines were cultured in DMEM (Invitrogen) supplemented with 10% fetal calf serum, 100 U/ml penicillin-streptomycin and 2 mM L-glutamin (Invitrogen) and maintained at 37°C in 5% CO_2_.

Neurospheres: SVZ isolated from brains of five-day-old mice, were dissociated with trypsin-EDTA (Invitrogen) and cultured in a proliferation medium containing 75% DMEM, 25% F12 Ham (Invitrogen), ITS additive (insulin, transferrin, selenite; Invitrogen), transferrin (100 µg/ml; Sigma), progesterone (20 nM; Sigma), penicillin/streptomycin (100 U/ml; Invitrogen) supplemented with bFGF (basic fibroblast growth factor, 10 ng/ml; Peprotech), EGF (epidermal growth factor, 10 ng/ml; Peprotech) and B27 additive (1/50; Invitrogen) and maintained at 37°C in 5% CO2.

### Flow Cytometry

3×10^5^ cells were stained for 15 min on ice with either anti-mouse pan-RAE-1 (R&D systems, clone 186107), anti-mouse RAE-1ε (R&D systems; clone 205001), anti-mouse RAE-1δ (R&D systems; clone 199205), isotype controls or the mouse recombinant NKG2D/Fc chimera (R&D systems) diluted in PBS 1% FCS, 0.02% sodium azide and then incubated with PE labeled anti-rat antiserum (Jackson ImmunoResearch Laboratories, Inc.) or with PE labeled anti-human antiserum (Beckman Coulter). The cells were washed and fixed with 2% paraformaldehyde (Sigma) in PBS EDTA 5 mM and analyzed on FACSCanto (Becton Dickinson). The data were analyzed with the CellQuest software (Becton Dickinson).

### RNA extractions, RT-PCR and –qPCR

Tissues were dissected, weighted and placed in RNALater solution (Qiagen, CA). Total RNA extraction of tissues and cell lines were performed with the RNeasy Mini Kit (Qiagen). The reverse transcription was performed with M-MLV reverse transcriptase (Invitrogen). All primers are reported in Table S1. PCR were performed on a T1 Thermocycler (Biometra) with the Taq DNA Polymerase Recombinant (Invitrogen). The amplification procedure was done as follows: 2 min. denaturation at 95°C, 40 cycles with 30 sec. at 95°C, 30 sec. of annealing at the appropriate temperature and 30 sec. elongation at 72°C, and finally 10 minutes elongation at 72°C. The PCR products were loaded on 2% agarose gels containing SYBRSafe (Invitrogen). Quantitative PCR was performed on Stratagene's MX3000P QPCR System with the Brilliant SYBR Green QPCR Master Mix (Stratagene). The amplification procedure included a 10 minutes step at 95°C followed by 40 cycles with 30 sec. at 95°C and 1 min at 62°C and Melting Curve analysis. Results were expressed relatively to GAPDH as endogenous control. For circadian rhythm studies we used 36B4 as control [10].

### Olfactory axotomy

Eight-week-old female mice were anesthetized with intraperitoneal injections of Xylasin (10 µg/g body weight, Ronpum 2%, Bayer) and Ketamin (0.1 mg/g body weight, Kétamine 1000, Virbac). An incision was made on the head along the midline of the scalp. A craniotomy was bilaterally performed over the anterior part of the olfactory bulbs and the axons of the olfactory nerves were cut with a needle. Sham-operated controls were subjected to the same procedure but the axons were not sectioned. Then, the skin was sutured and animals were sacrificed at Day 6 for analysis.

### Experimental Autoimmune Encephalomyelitis

Eight-week-old female mice were immunized by injecting subcutaneously 200 µl of an emulsion containing 200 µg of 35–55 myelin oligodendrocyte glycoprotein (MOG) peptide (>95% pure, Neosystem) in complete Freund adjuvant (CFA) (DIFCO) supplemented with 400 µg of H37Ra Mycobacterium tuberculosis (DIFCO). 50 ng of Bordetella pertussis toxin (Sigma) in 100 µl of PBS were injected intra-peritoneously at Day 0 and Day 1. The animals were blindly monitored daily by weighing and clinical scoring on a scale of 0 to 6 with the following criteria: 0: no deficit; 1: limp tail; 2: mild hind leg weakness; 3: half-paralysis of hind leg; 4: total hind leg paralysis; 5: fore leg and hind leg paralysis; 6: moribund. Animals were sacrificed at Day 30, during the chronic phase of the disease.

### 5′RACE

Enrichment of polyA+ RNA from total RNA was performed using the Qiagen oligotex mini kit according to the manufacturer's protocol. The 5′ RACE was carried out with the 5′-full RACE Core Set kit (Takara) as follows: first, a reverse transcription using the AMV reverse transcriptase XL was done with a 5′ phosphorylated primer (Table S1), then 1 h RNaseH treatment at 30°C was performed followed by a 12 h ligation with T4 DNA ligase at 15°C. The unknown part of the sequence was amplified in a first PCR with a primer couple S1A1 followed by a nested PCR with primer couple S2A2 (Table S1). The final PCR products were inserted into the pCRII-TOPO vector (Invitrogen) and sent for sequencing (Genome Express).

### Cloning methods and strategies

Promoter cloning. Genomic DNA was obtained from mouse C57BL/6 liver with the Qiagen DNeasy blood and tissue kit. Genomic DNA was amplified 1,500 bp upstream of exon 1 with primers containing restriction sites for XhoI and HindIII (Table S1) and the PrimeStar DNA polymerase (Lonza). Genomic DNA upstream of exon 3b (5,651 bp for *rae-1δ* and 5,474 bp for *rae-1ε*) was amplified with primers containing restriction sites for KpnI and NheI and the PrimeStar DNA polymerase. The PCR products were digested and joined in the pGL3 Basic Luciferase reporter vector (Promega) with the T4 DNA ligase (Invitrogen). We used as control the pGL3-C plasmid which contains the SV40 promoter.

Rae-1 cloning. The vector pEGFP-C1 (Clontech) was used to express EGFP and to insert Rae-1 coding sequences after the removal of the EGFP sequence between the NheI and BamHI restriction sites. Rae-1δ, Rae-1ε and Rae-1γ transcripts were amplified from cDNA with primers containing NheI and BamHI sites and the PrimeStar DNA polymerase (Lonza). The PCR products were digested and joined in the vector with the T4 DNA ligase. In all cases, the constructs were verified by sequencing (Genome Express). For transfection, DNA was purified with the Plasmid Purification kit for isolation of ultrapure, transfection grade plasmid DNA (Qiagen).

### Cell transfection and luciferase assay

Cells were transfected using JetPEI (Polyplus-transfection) with the firefly luciferase reporter plasmids containing *rae-1* promoters. Transfection efficiency was assessed by the co-tranfection of pRL-null plasmid (Promega) encoding *Renilla* luciferase. The luciferase activity was measured with the Dual Luciferase Reporter assay (Promega) on a Lumat LB 9507 (Berthold Technologies).

## Supporting Information

Table S1 List of primers used for the different experiments of PCR, quantitative PCR, 5′ RACE and cloning strategies.
(0.04 MB XLS)

Figure S1 Sequence of PCR products from liver after amplification with exon 5 and exon 9 primers. We observed the superimposition of two sequences, one corresponding to Rae-1δ and the other, less represented, and corresponding to Rae-1ε.
(0.57 MB TIF)

Figure S2 NK cells are recruited in lumbar spinal cord after EAE. A, Spinal cords were dissected 25 days after EAE induction and NKp46 transcripts were quantified. Results were expressed as mean of 3 analysis +/- SEM relatively to GAPDH as endogenous control. Expression in healthy spinal cords (n = 3) was compared with expression in spinal cords from 3 mice suffering mild EAE (clinical score 2) and 3 mice suffering severe EAE (clinical score 4). B, Lymphocytes were isolated from spinal cord, 25 days after EAE induction. The expression of NKG2D protein was analyzed by flow cytometry in NK cells (blue population) and CD8 lymphocytes (green population) another cell population able to express NKG2D.
(0.65 MB TIF)

Figure S3 Comparison of RAE-1 sequences and illustration of the exposed loop of RAE-1γ,δ and ε. A, Amino acid sequences of RAE-1αδ, β, γ, δ and ε were aligned using ClustalW. Secondary structure elements are identified (black arrows for β-sheets, and white cylinders for α-helix) based on RAE-1β three-dimensional structure (PDB entry 1JFM. The PLWY motif (localized between sheets β-1 and β-2) was highlighted in red for all five sequences. B, Ribbon diagrams of RAE-1γ, δ and ε were obtained by replacing amino acids from the crystal structure of RAE-1β (PDB entry 1JFM) and using the SWISS-MODEL server to generate the calculated structure of each isoform. On the left panel the ‘KDPTPADPLWY’ loop (between sheets β-1 and β-2) was highlighted in purple, Trp51 previously involved in ‘RAE-1-NKG2D’ recognition is shown in yellow, and Tyr52 in orange. In the central panel, RAE-1δ exhibits a shorter loop due to the ‘PLWY’ deletion. In RAE-1ε, right panel, mutations (i.e. ‘LPWC’) do not alter the structure of this loop. Trp51 (yellow) and Cys52 (red) are shown as stick representations superposed on the ribbons. The structures are visualized using Pymol software (The PyMOL Molecular Graphics System, Version 1.2r3pre, Schrödinger, LLC, http://www.pymol.org/).
(1.71 MB TIF)
