# Supplementary figures and images for: The NKG2D Ligands RAE-1δ and RAE-1ε Differ with Respect to Their Receptor Affinity, Expression Profiles and Transcriptional Regulation
Source: PLoS One. 2010 Oct 19;5(10):e13466. doi: 10.1371/journal.pone.0013466 (PMC2957426; doi:10.1371/journal.pone.0013466)

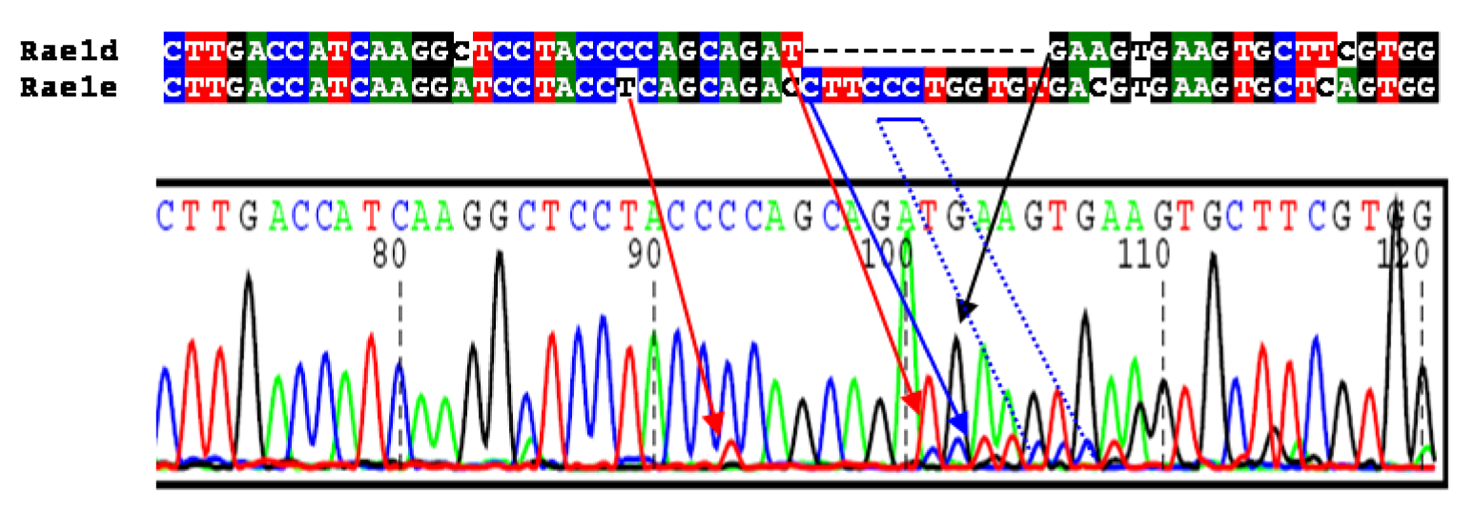

Supplement: Figure S1 — Sequence of PCR products from liver after amplification with exon 5 and exon 9 primers. We observed the superimposition of two sequences, one corresponding to Rae-1δ and the other, less represented, and corresponding to Rae-1ε. (0.57 MB TIF) [file pone.0013466.s002.tif]

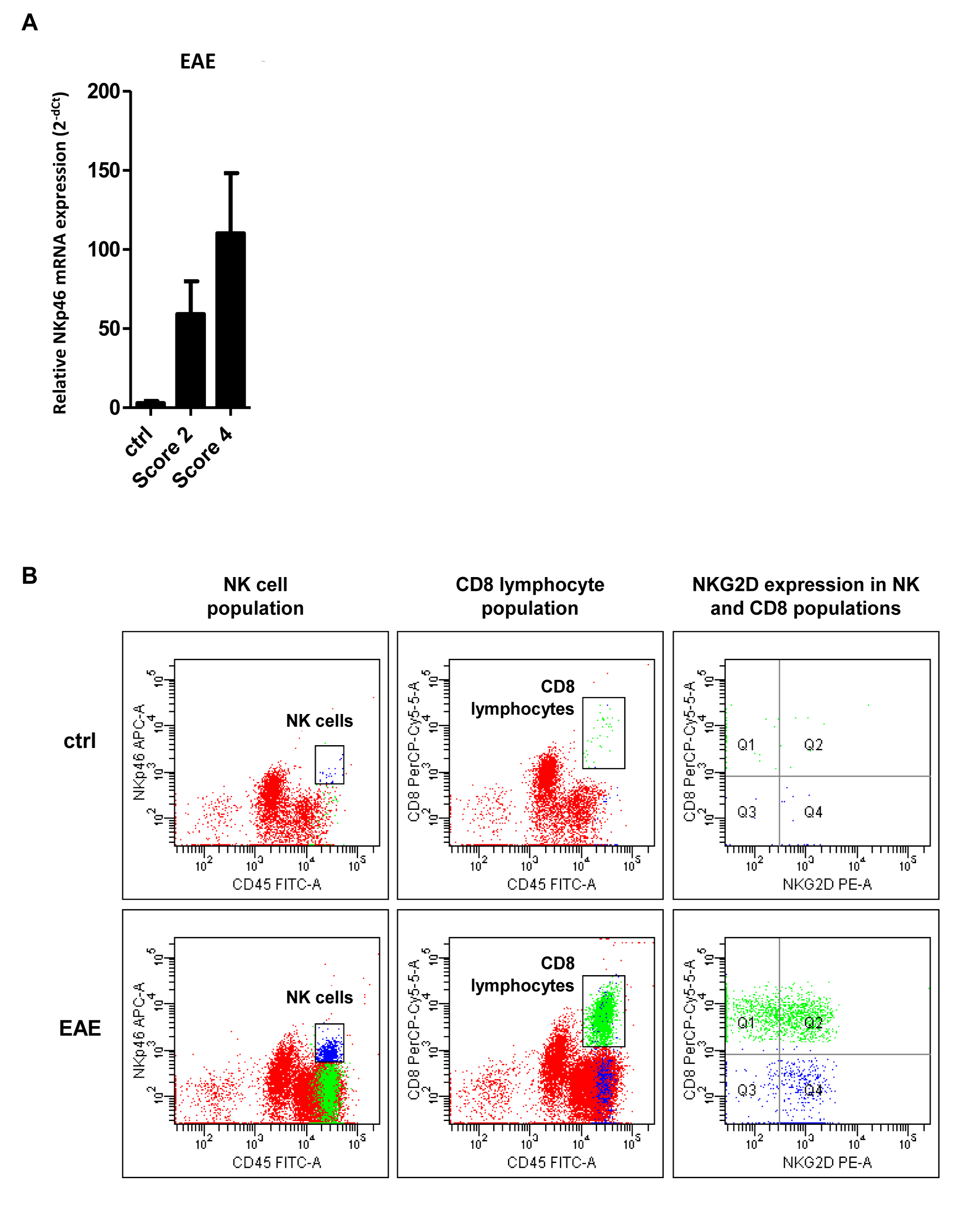

Supplement: Figure S2 — NK cells are recruited in lumbar spinal cord after EAE. A, Spinal cords were dissected 25 days after EAE induction and NKp46 transcripts were quantified. Results were expressed as mean of 3 analysis +/- SEM relatively to GAPDH as endogenous control. Expression in healthy spinal cords (n = 3) was compared with expression in spinal cords from 3 mice suffering mild EAE (clinical score 2) and 3 mice suffering severe EAE (clinical score 4). B, Lymphocytes were isolated from spinal cord, 25 days after EAE induction. The expression of NKG2D protein was analyzed by flow cytometry in NK cells (blue population) and CD8 lymphocytes (green population) another cell population able to express NKG2D. (0.65 MB TIF) [file pone.0013466.s003.tif]

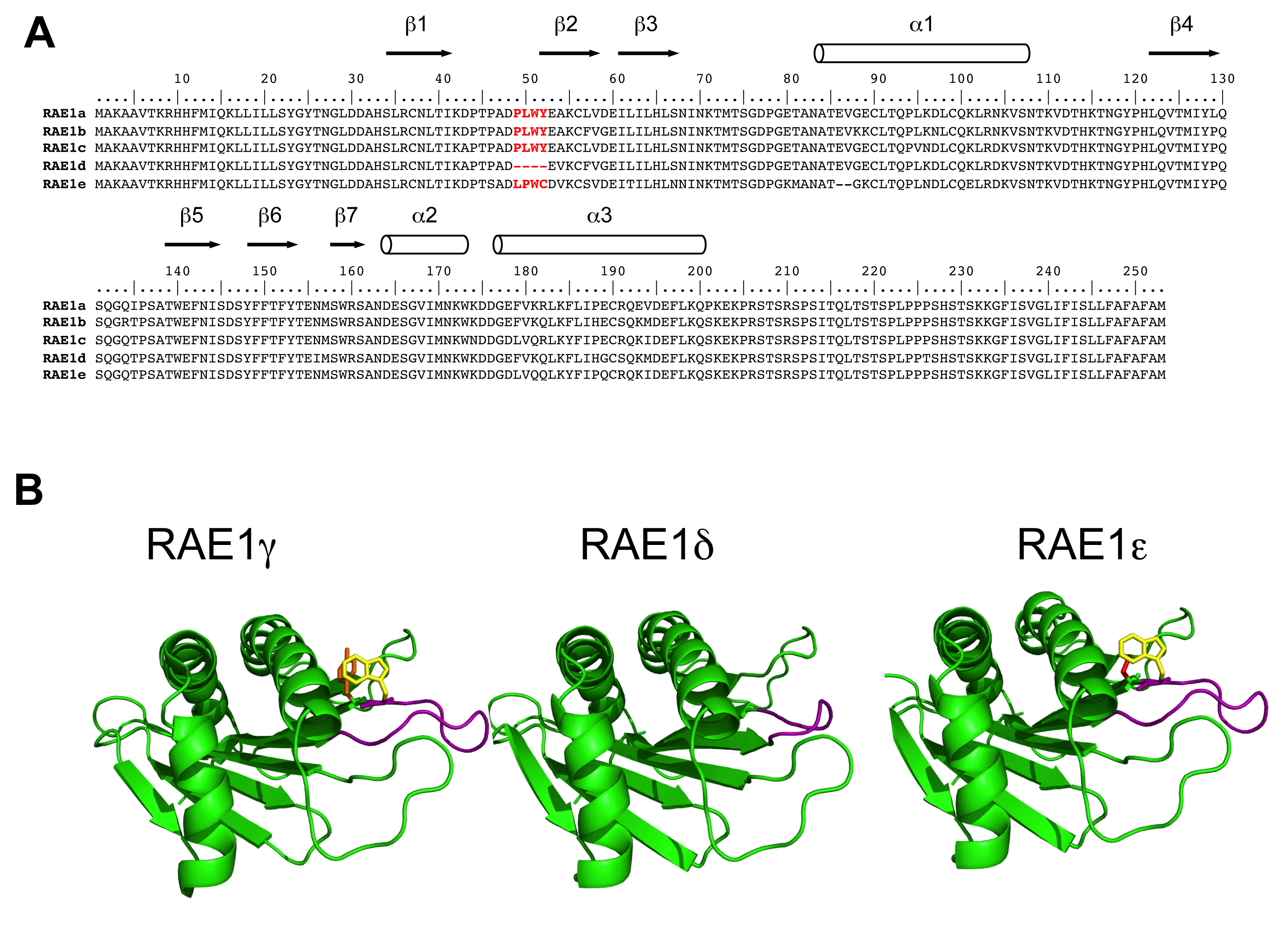

Supplement: Figure S3 — Comparison of RAE-1 sequences and illustration of the exposed loop of RAE-1γ,δ and ε. A, Amino acid sequences of RAE-1αδ, β, γ, δ and ε were aligned using ClustalW. Secondary structure elements are identified (black arrows for β-sheets, and white cylinders for α-helix) based on RAE-1β three-dimensional structure (PDB entry 1JFM. The PLWY motif (localized between sheets β-1 and β-2) was highlighted in red for all five sequences. B, Ribbon diagrams of RAE-1γ, δ and ε were obtained by replacing amino acids from the crystal structure of RAE-1β (PDB entry 1JFM) and using the SWISS-MODEL server to generate the calculated structure of each isoform. On the left panel the ‘KDPTPADPLWY’ loop (between sheets β-1 and β-2) was highlighted in purple, Trp51 previously involved in ‘RAE-1-NKG2D’ recognition is shown in yellow, and Tyr52 in orange. In the central panel, RAE-1δ exhibits a shorter loop due to the ‘PLWY’ deletion. In RAE-1ε, right panel, mutations (i.e. ‘LPWC’) do not alter the structure of this loop. Trp51 (yellow) and Cys52 (red) are shown as stick representations superposed on the ribbons. The structures are visualized using Pymol software (The PyMOL Molecular Graphics System, Version 1.2r3pre, Schrödinger, LLC, http://www.pymol.org/). (1.71 MB TIF) [file pone.0013466.s004.tif]
